# Insights Into the Photocatalytic Arene Bromination Enabled by Cs_2_AgBiBr_6_ Microparticles

**DOI:** 10.1002/chem.70837

**Published:** 2026-02-28

**Authors:** Daniele Conelli, Chiara Lo Porto, Mokurala Krishnaiah, Kimmo Lahtonen, G. Krishnamurthy Grandhi, Nicola Margiotta, Paola Vivo, Gian Paolo Suranna, Roberto Grisorio

**Affiliations:** ^1^ Dipartimento di Ingegneria Civile, Ambientale, del Territorio, Edile e di Chimica (DICATECh) Politecnico di Bari via Orabona, 4 Bari 70125 Italy; ^2^ Department of Life Science, Health, and Health Professions Università degli Studi Link Roma Italy; ^3^ Hybrid Solar Cells, Faculty of Engineering and Natural Sciences, FI‐33014 Tampere University Tampere Finland; ^4^ Dipartimento di Chimica Università degli Studi di Bari “Aldo Moro” Bari Italy; ^5^ CNR‐NANOTEC – Institute of Nanotechnology C/O Campus Ecoteckne Lecce Italy

**Keywords:** bromination, double perovskite, heterogeneous catalysis, photocatalysis, radical pathway

## Abstract

The development of sustainable halogenation strategies remains a central challenge in synthetic chemistry, particularly for the preparation of aryl bromides, which are key intermediates in pharmaceuticals, materials, and cross‐coupling transformations. Here, we report an atom‐efficient photocatalytic bromination of electron‐rich arenes enabled by a low‐toxicity Ag–Bi double perovskite microparticles. Under mild conditions and using hydrobromic acid as a bromine source, the photocatalyst promotes highly selective mono‐bromination of 1,3,5‐trimethoxybenzene with excellent yields (up to 95.4%) and superior performance compared to the benchmark perovskite‐like materials, such as CsPbBr_3_ and Cs_3_Bi_2_Br_9_. Mechanistic investigations reveal an unprecedented activation mode of the transformation, in which bromine radicals originate from the Cs_2_AgBiBr_6_ surface, as demonstrated by radical‐trapping studies and catalyst degradation observed in the absence of HBr. Control experiments further rule out the involvement of oxidant species, confirming that surface‐derived bromine radicals are regenerated by external bromide. The photocatalyst maintains activity over multiple cycles, retaining its structural integrity with only minor AgBr formation upon five consecutive runs. The method displays broad substrate scope, encompassing anisole derivatives, polymethoxylated arenes, and heteroaromatics, with regioselectivity governed by substrate properties. Overall, these findings highlight the unique reactivity of double‐perovskite surfaces and open new avenues for sustainable photocatalytic halogenation chemistry.

## Graphical abstract

A sustainable photocatalytic bromination of electron‐rich arenes is enabled by low‐toxicity Cs_2_AgBiBr_6_ double‐perovskite microparticles using HBr as an atom‐economical bromine source. Surface‐derived bromine radicals drive highly selective mono‐bromination under mild conditions. The catalyst outperforms benchmark perovskites, operates via a unique lattice‐bromide activation mechanism, and maintains structural integrity upon recycling.

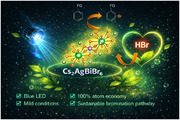

## 1 Introduction

**FIGURE 1:**
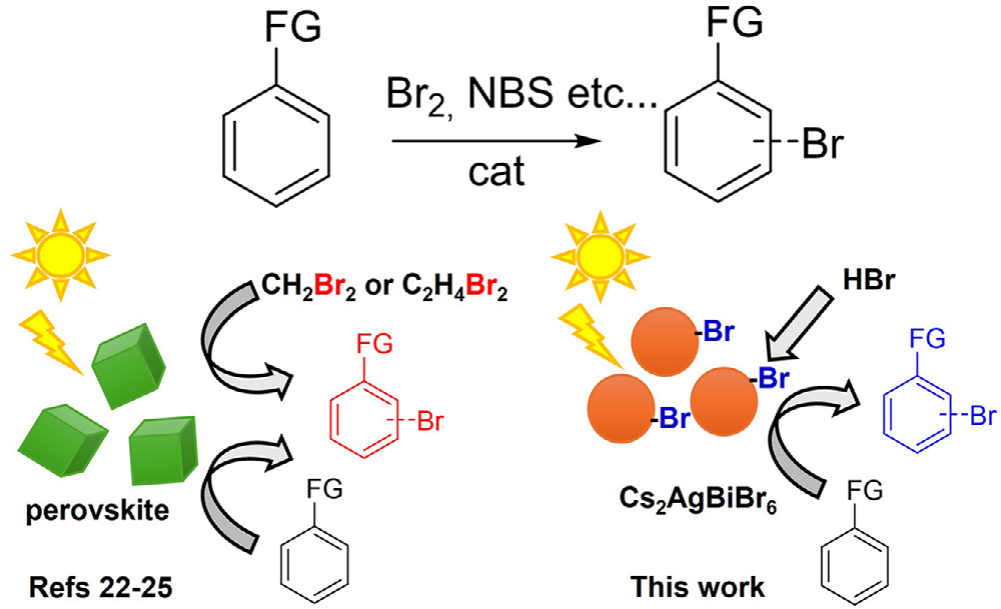
Synthetic strategies for the bromination of aryl substrates (FG = functional group). Comparison between approaches employed in this work and those reported in literature (left).

Aromatic compounds bearing bromine atoms are widely employed in pharmaceuticals (e.g., brotizolam, merbromin) as well as in advanced materials (such as Eosin Y) [1, 2]. At the same time, aryl bromides serve as essential coupling partners in numerous metal‐catalyzed cross‐coupling reactions, enabling the efficient construction of complex molecules used across a broad range of applications [3, 4, 5, 6, 7]. Owing to the ubiquity and synthetic utility of these motifs, the predictable and selective collocation of bromine into aromatic scaffolds under mild and practical conditions continues to attract considerable interest. Traditionally, arene bromination has been achieved through Friedel/Crafts‐type electrophilic aromatic halogenation [8], Sandmeyer‐type transformations of diazonium salts [9] or transition‐metal‐catalyzed strategies [10]. Among these methods, electrophilic aromatic halogenation remains the most convenient and widely employed approach. However, it typically relies on the use of elemental bromine under harsh conditions, electrophilic Br^+^ donors (including N‐halo reagents), or bromide sources combined with hazardous and/or corrosive oxidants. Although milder precursors such as N‐bromosuccinimide are frequently used, they still suffer from poor atom economy (Figure 1) [11]. Despite advances in catalytic protocols, many established methods continue to generate environmentally detrimental byproducts [12, 13]. These challenges accentuate the need for alternative bromine sources and efficient catalytic systems capable of enabling regio‐ and chemoselective bromination under sustainable and operationally simple reaction conditions.

Photocatalysis has emerged as a powerful strategy to enhance the efficiency, selectivity, and sustainability of aromatic bromination, because significantly reduces chemical waste and utilizes a clean energy source [14]. In this context, halide perovskite and perovskite‐inspired materials have gained significant attention due to their exceptional optoelectronic properties, including high absorption coefficients, tunable band gaps, and excellent charge carrier mobility and lifetimes [15, 16, 17, 18, 19, 20, 21]. The predominant strategies for bromination employ alkyl bromides as halide sources, activated photocatalytically via a reductive pathway (Figure 1) [22, 23, 24, 25]. While mechanistically consistent with the slow release of activated halides to warrant high selectivity to the process [26, 27], this approach fails to address the inherent toxicity of the halide source and shows limited atom economy.

In this study, we present a sustainable and atom‐efficient strategy for the selective bromination of electron‐rich arenes, achieved under mild conditions using a low‐toxicity halide perovskite heterogeneous photocatalyst. By employing hydrobromic acid (HBr) as a less hazardous bromine source, we demonstrate that Cs_2_AgBiBr_6_ microparticles display superior catalytic activity compared to established perovskite‐like materials (CsPbBr_3_ and Cs_3_Bi_2_Br_9_). This enhanced performance stems from an unprecedented mechanism in which bromine atoms located on the catalyst surface (Figure 1) act as halide donors, while the external HBr source replenishes the surface atoms that are lost. Overall, this work opens new avenues for expanding the potential of perovskite‐based photocatalysts in surface‐promoted transformations.

## 2 Results and Discussion

**FIGURE 2:**
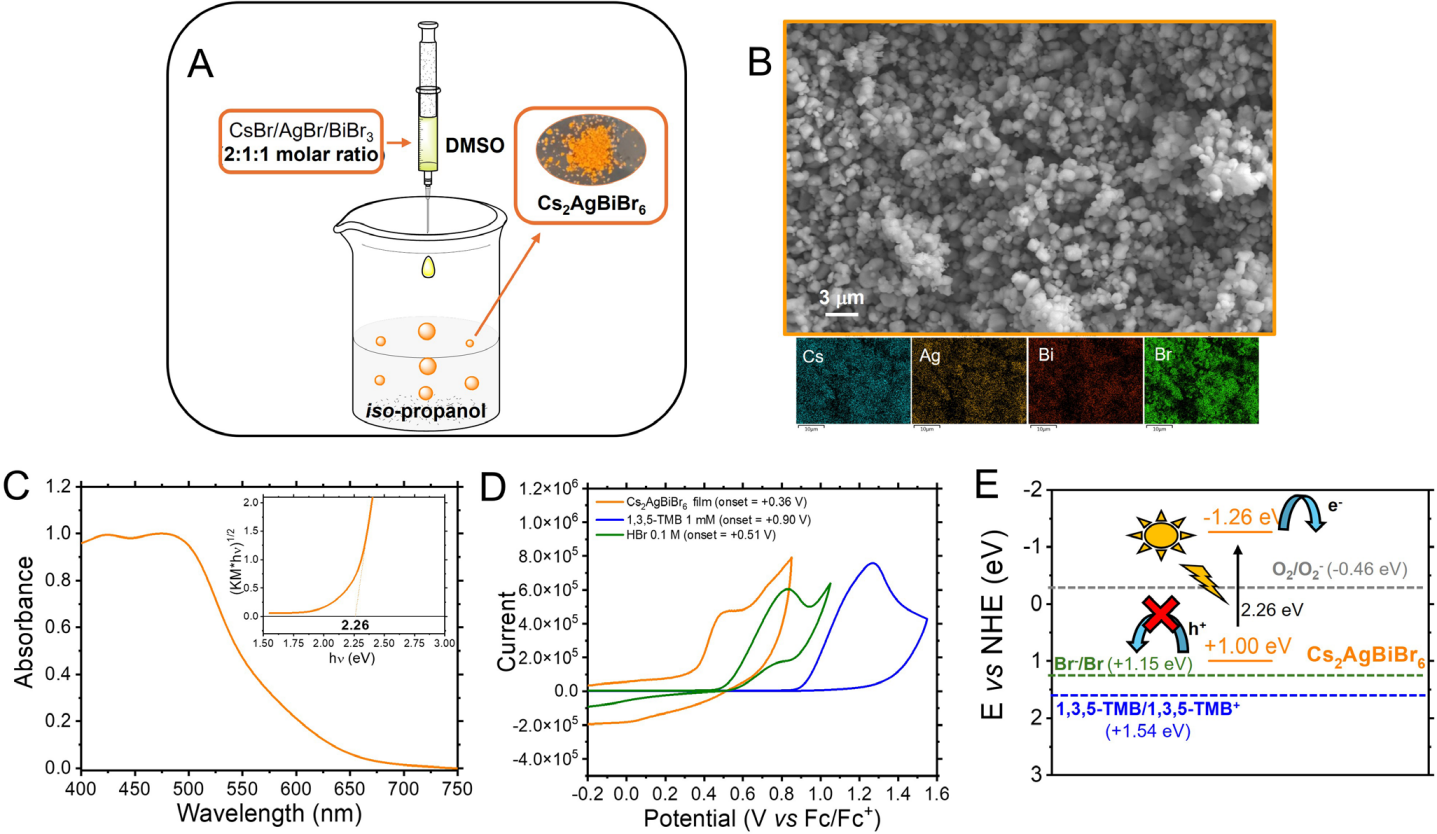
(A) Schematic representation of the synthetic methodology for obtaining Cs_2_AgBiBr_6_ microparticles. (B) A representative SEM image of the Cs_2_AgBiBr_6_ sample, accompanied by elemental distribution maps for Cs, Ag, Bi, and Br. (C) Diffuse reflectance spectrum of the Cs_2_AgBiBr_6_ sample. Inset: Corresponding Kubelka–Munk plot used for indirect bandgap estimation. (D) Cyclic voltammetry of Cs_2_AgBiBr_6_ film, HBr, and TMB. (E) Energy levels (vs NHE) of the reaction components, indicating that only photoinduced electron transfer to molecular oxygen is feasible with Cs_2_AgBiBr_6_ microparticles.

The Cs_2_AgBiBr_6_ microparticles were prepared via antisolvent reprecipitation methods, similarly to the process used in previous studies for obtaining CsPbBr_3_ and Cs_3_Bi_2_Br_9_ [28, 29]. In this approach, cesium, bismuth, and silver halides were combined in appropriate molar ratios in highly polar solvents such as dimethyl sulfoxide (DMSO), followed by rapid injection into a less polar medium, *iso*‐propanol, to induce the rapid crystallization of the double‐perovskite structure, evidenced by the formation of an orange precipitate (Figure 2A). Scanning electron microscopy (SEM) coupled with energy‐dispersive X‐ray spectroscopy (EDX) revealed that the Cs_2_AgBiBr_6_ particles possess a uniform quasi‐spherical morphology and exhibit a homogeneous distribution of Cs, Br, Bi, and Ag elements (Figure 2B).

**FIGURE 3:**
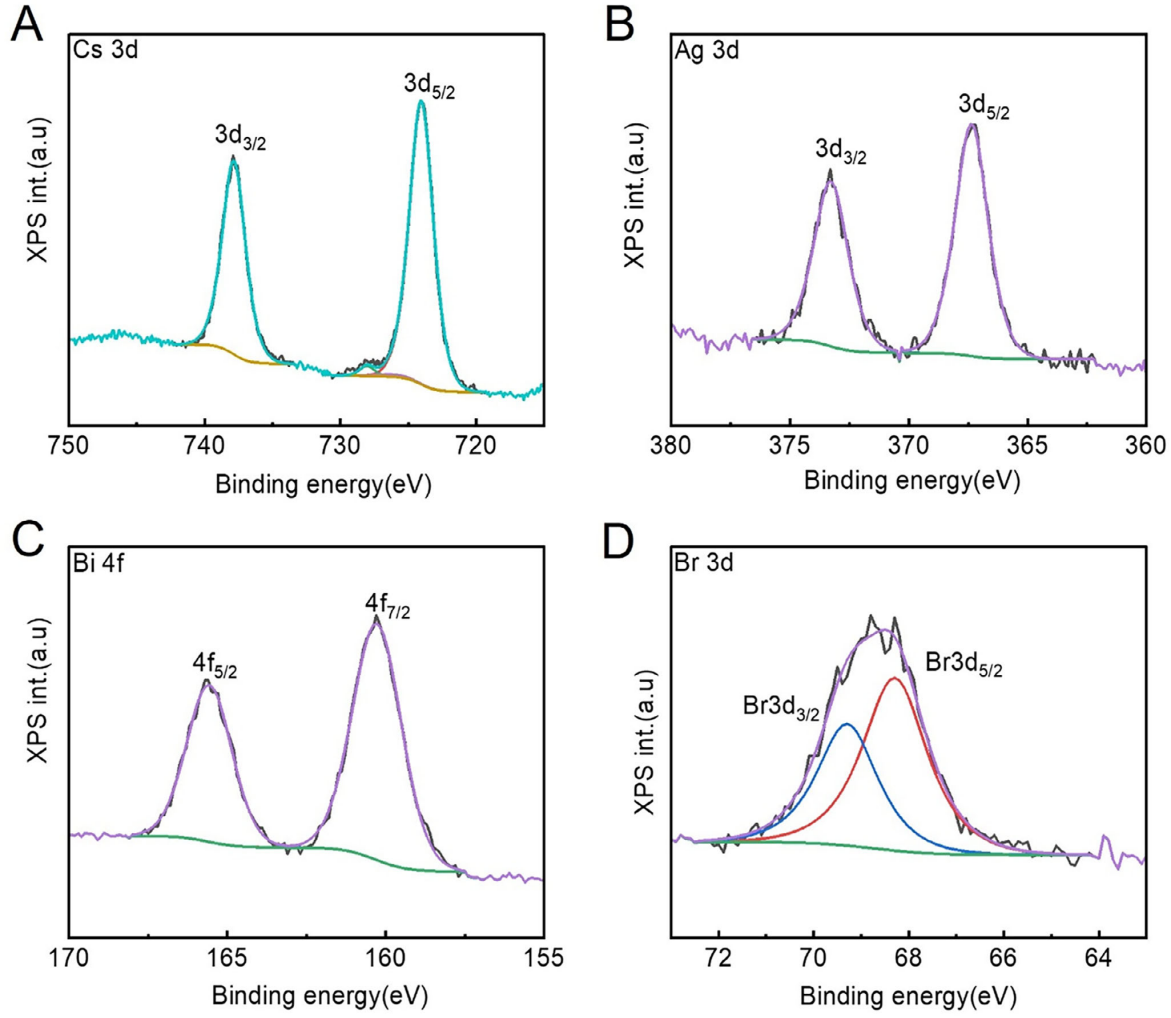
High‐resolution XPS spectra of (A) Cs 3d, (B) Ag 3d, (C) Bi 4f, and (D) Br 3d for the prepared Cs_2_AgBiBr_6_ samples.

X‐ray photoelectron spectroscopy (XPS) was employed to determine the elemental composition and oxidation states of the synthesized Cs_2_AgBiBr_6_ particles. The survey spectra, presented in Figure S1, confirm the presence of cesium, silver, bismuth, bromine, and carbon, with the fitted C 1s spectrum shown in Figure S1. High‐resolution XPS spectra for Cs 3d, Ag 3d, Bi 4f, and Br 3d were analyzed using Gaussian–Lorentzian peak fitting, revealing well‐defined spin‐orbit doublets for each element, as illustrated in Figure 3. The Cs 3d spectrum exhibited peaks at 737.90 eV (3d_3/2_) and 724.04 eV (3d_5/2_), indicating the presence of monovalent Cs^+^, while a small satellite peak around 728 eV may be attributed to the use of a nonmonochromatized X‐ray source. Similarly, the Ag 3d peaks at 373.30 eV (3d_3/2_) and 367.36 eV (3d_5/2_) correspond to monovalent Ag^+^. The Bi 4f spectrum displayed peaks at 163.70 eV (4f_5/2_) and 158.37 eV (4f_7/2_), confirming the presence of trivalent Bi^3^^+^. The Br 3d spectrum showed peaks at 69.29 eV (3d_3/2_) and 68.29 eV (3d_5/2_), consistent with monovalent Br^−^, with no evidence of additional oxidation states. Overall, the XPS analysis indicates that only a single oxidation state was detected for Cs, Ag, Bi, and Br, as all core‐level peaks (4f_7/2_, 4f_5/2_, 3d_5/2_, 3d_3/2_, etc.) exhibited a single symmetric component. The presence of multiple peaks, such as more than one 4f_7/2_ peak, would suggest multiple oxidation states or bonding environments, which were not observed in this case. The observed binding energies align with literature values for Cs_2_AgBiBr_6_, confirming the expected oxidation states for the elements [30, 31]. Quantitative analysis yielded an atomic composition of 25.45% (*vs* 28.42% of EDX) Cs, 9.15% (*vs* 18.91% of EDX) Ag, 12.60% (*vs* 7.62% of EDX) Bi, and 52.80% (*vs* 45.07% of EDX) Br, suggesting an enrichment in the halide at the microparticle surface.

The UV‐vis diffuse reflectance spectrum revealed a broad band absorption profile in the visible regime for the Cs_2_AgBiBr_6_ microparticles (Figure 2C). The corresponding Kubelka–Munk fit, assuming an indirect bandgap transition, indicated a bandgap of 2.26 eV (inset of Figures 2C and S2). The combination of efficient light harvesting and an indirect band structure in Cs_2_AgBiBr_6_ microparticles is particularly advantageous for photocatalytic applications, because strong absorption enables effective photon utilization, while the indirect character enhances charge separation by suppressing rapid electron–hole recombination [32].

To benchmark the photoinduced oxidative threshold of the photocatalyst, the electron‐rich 1,3,5‐trimethoxybenzene (TMB) was chosen as the model substrate. Cyclic voltammetry (used to estimate the oxidation potential of the reaction components) showed an irreversible oxidation event for TMB (onset at +0.90 V vs. Fc/Fc^+^ reference) in acetonitrile, while HBr exhibited a lower oxidation potential (onset at +0.51 V vs. Fc/Fc^+^ reference), as reported in Figure 2D. The Cs_2_AgBiBr_6_ film showed a lower oxidation onset at +0.36 V vs. Fc/Fc^+^, indicating that photoinduced hole transfer from the photoexcited catalyst to both HBr and TMB is unlikely. Bandgap energy defines the band edges with redox potentials, demonstrating that the only species photoinductively activable from the Cs_2_AgBiBr_6_ microparticles is molecular oxygen with the formation of the corresponding superoxide (Figures 2E and S3).

However, trusting in an alternative photoinduced generation of reactive species promoted by Ag(I)‐based perovskites, the photocatalytic activity of Cs_2_AgBiBr_6_ microparticles and the role of HBr as a bromine source was anyway probed with the electron‐rich TMB as a model transformation (entry 1 of Table 1). The reaction was performed under ambient conditions (open vessel, air atmosphere) using blue LED irradiation as the sole energy input. Complete substrate conversion was achieved after 12 h, affording the mono‐brominated product with 95.4% yield (Figure S4) and excellent regioselectivity. The product evolution was found to be 1060 µmol g^−^^1^ h^−^^1^, a value comparable to that proposed in literature for different photocatalysts and bromine sources [22, 23, 24, 25].

**TABLE 1:** Screening and control experiments for the bromination of 1,3,5‐trimethoxybenzene (TMB).

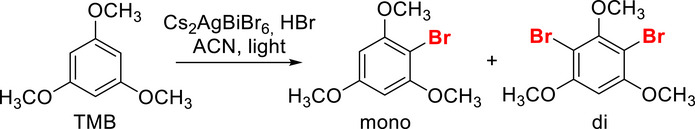					
**entry**	**variation**	**time**	**conv. (%)** ^a^	**yield (%)** ^a^	**mono:di**
1	—	12	100.0	95.4	100:0^b^
2	w/o Cs_2_AgBiBr_6_	24	—	—	—
3	dark	24	—	—	—
4	w/o HBr	24	21.0	18.8	100:0^a^
5	N_2_	24	82.5	81.8	100:0^a^
6	CsPbBr_3_	12	57.1	44.9	79:21^a^
7	Cs_3_Bi_2_Br_9_	12	60.0	51.1	85:15^a^
8	CPME	12	67.6	42.2	100:0^a^
9	THF	12	29.1	23.7	100:0^a^
10	DCM	12	46.6	46.6	100:0^a^
11	EtOAc	12	100.0	91.3	100:0^a^

Standard conditions: TMB (0.04 mmol), HBr (0.05 mmol), acetonitrile (3 mL), and Cs_2_AgBiBr_6_ (3 mg, 6% mol/mol). Reactions were conducted in air with a blue LED (3 W) light source.

^a^ Determined by GC‐MS using biphenyl as an internal standard (results are based on three replicates).

^b^ Determined by ^1^H NMR.

To clarify the role of individual components in the photocatalytic bromination of TMB, a series of control experiments were conducted under different conditions (Table 1). In the absence of the photocatalyst (entry 2 of Table 1), no detectable product formation was observed, confirming that HBr and TMB alone are insufficient to drive the reaction. Both HBr and TMB are photochemically inert under the applied conditions, highlighting the essential role of Cs_2_AgBiBr_6_ in initiating the photocatalytic reaction. Similarly, no substrate conversion occurred in the dark (entry 3 of Table 1), indicating that light is required to activate the reaction components.

Conversely, in the absence of HBr (entry 4 of Table 1), a moderate substrate conversion (21.0%) and product formation (18.8%) were observed, providing mechanistic relevant insights alongside the role of HBr in the reaction. This result implies that Cs_2_AgBiBr_6_ can promote bromination via intrinsic halide release under irradiation, being the sole bromine source in the reaction conditions reported in entry 4 of Table 1. The concurrent degradation of the photocatalyst (a white solid is recovered after reaction) supports this hypothesis, as bromine donation from the double‐perovskite lattice likely compromises its structural integrity, thereby limiting catalytic turnover (18.8% yield) in the absence of an external bromine source (HBr). X‐ray diffraction (XRD) analysis of the degraded material recovered from the reaction conducted without HBr reveals extensive evolution of AgBr from the catalyst (Figure S5). Furthermore, this reaction pathway is fully consistent with the redox potentials of the involved species (photocatalyst, HBr, and TMB) of the synthetic protocol, as indicated in Figure 2E.

The reaction also proceeds under a nitrogen atmosphere (entry 5 of Table 1), indicating that molecular oxygen is not strictly required for the transformation. However, its presence appears to enhance the overall efficiency, suggesting that O_2_ plays a beneficial (though not essential) role in sustaining high photocatalytic performance through accepting photogenerated electrons (to form superoxide) and dissociating the photogenerated charges.

Importantly, the Ag‐Bi double perovskite exhibited superior photocatalytic performance and selectivity compared to the CsPbBr_3_ and Cs_3_Bi_2_Br_9_ counterparts (entries 1 vs. 6–7 of Table 1). This reduced activity is observed notwithstanding the favorable energy levels of the CsPbBr_3_ and Cs_3_Bi_2_Br_9_ materials [24, 29], which in principle enable the photo‐activation of both bromide anions and TMB. Additionally, they are unable to promote the transformation in the absence of HBr indicating that the bromine donation from the catalyst is a prerogative of the double Ag–Bi perovskite.

Subsequently, the influence of the reaction medium on product formation was investigated (entries 8–11 of Table 1), revealing that neither ethereal nor halogenated solvents enhanced the conversion rate of the reaction. In contrast, ethyl acetate (with good environmental requisites as reaction medium) enabled complete substrate transformation within 12 h, affording yield and selectivity comparable to acetonitrile. These results indicate that the solvent polarity plays a crucial role in stabilizing the plausible radical and charged intermediates formed throughout the catalytic cycle.

To clarify the nature of the bromine species released by the catalyst in the reaction reported in entry 4 of Table 1 (i.e., in the absence of an external bromine source), we repeated the experiment in the presence of 2,2,6,6‐tetramethylpiperidine 1‐oxyl (TEMPO) as a radical scavenger. Under these conditions, no product formation was detected, indicating that the species initiating the catalytic cycle are bromine radicals originating from the catalyst surface. In a parallel experiment, the standard reaction (entry 1 of Table 1) was performed in the presence of TEMPO. Unexpectedly, GC–MS analysis revealed full substrate conversion after only 3 h of irradiation. This behavior can be rationalized by the reaction:

TEMPO·+HBr→TEMPO−H+Br·

In this scenario, TEMPO does not function as a radical scavenger; instead, it facilitates the generation of bromine radicals that accelerate the bromination process. This counterintuitive outcome provides additional support for a radical‐based reaction pathway.

**FIGURE 4:**
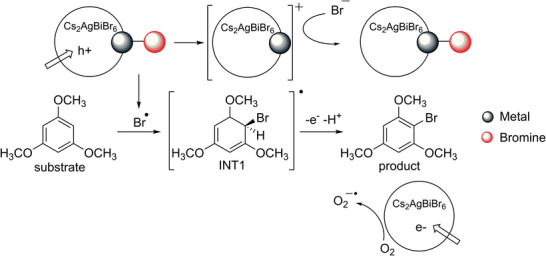
Plausible reaction pathway of the photocatalytic TMB transformation induced by Cs_2_AgBiBr_6_ microparticles.

Based on the gained information, we propose the reaction pathway outlined in Figure 4. Upon photoinduced exciton dissociation, the photogenerated hole promotes the homolytic breakage of the surface metal‐bromide bonds forming radical bromine attacking the aromatic scaffold generating the electron‐rich intermediate (INT1). The proton‐coupled electron transfer removes the hydrogen atom of the tetravalent carbon atom in INT1 repristinating the aromaticity of the compound. The catalyst surface is restored by the halide ions released from the reagent (HBr). The role of the superoxide (the only species thermodynamically capable of forming with the energy levels of the reaction components) is to facilitate photogenerated electron removal from the catalyst. Under anaerobic conditions, this action is in charge of the protons captures by the catalyst surface with relatively lower activity.

To rule out a possible role of oxidants (such as hydrogen peroxide) formed during catalysis in activating bromide toward a radical pathway [33], we qualitatively assessed H_2_O_2_ formation using a titanium–oxysulfate colorimetric test [34] under both the standard reaction conditions and in the absence of HBr (Figure S6). Indeed, alternative oxidative pathways (e.g., water oxidation) cannot be entirely excluded under our reaction conditions, which can lead to potential oxidants (hydrogen peroxide) of the bromides. However, our control experiments showed no detectable hydrogen peroxide in the reaction medium, indicating that bromine radicals are generated exclusively through the mechanism proposed in Figure 4.

**FIGURE 5:**
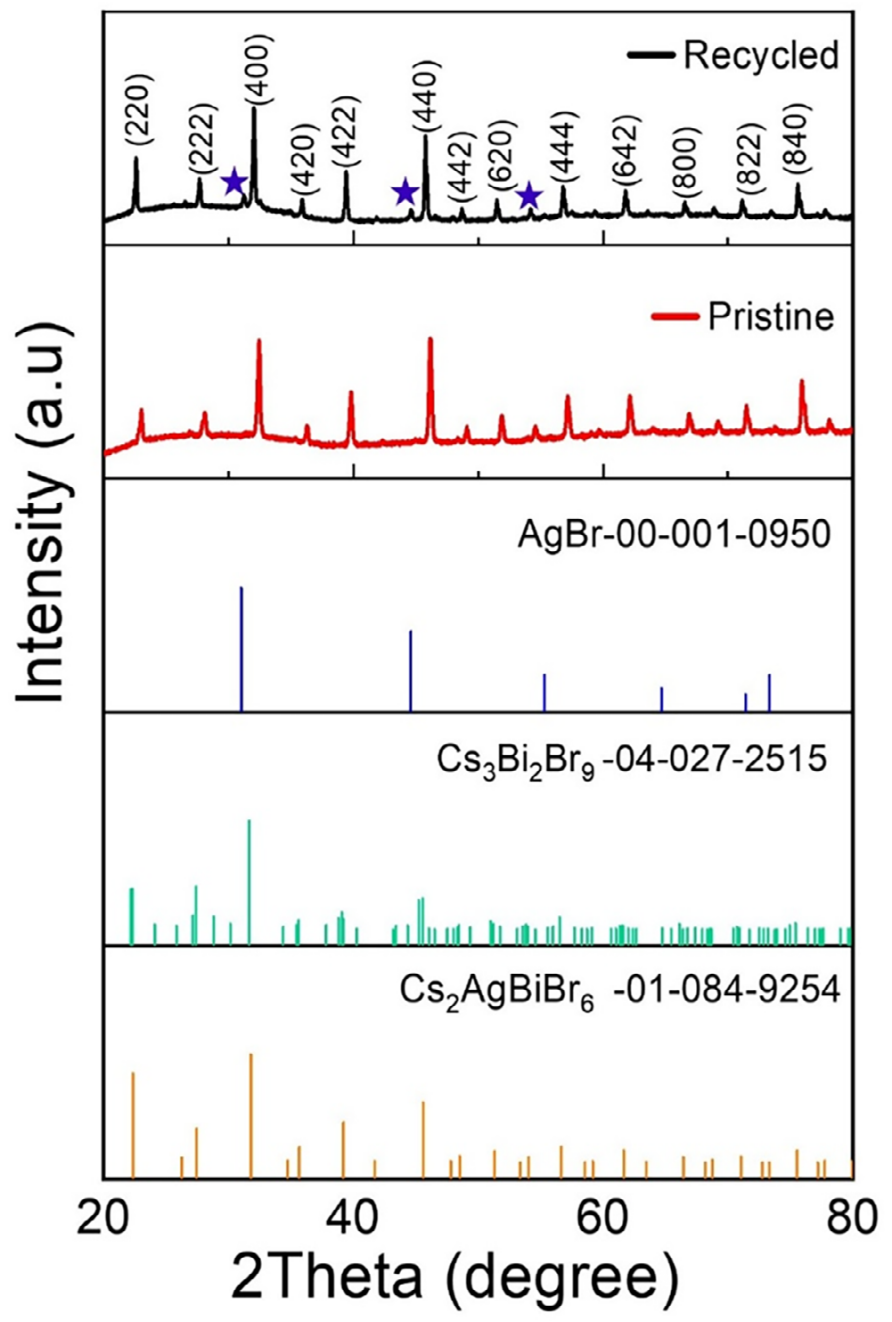
XRD analysis of pristine and recycled Cs_2_AgBiBr_6_ samples in comparison with the tabulated patterns of the relevant standards considering Cs_3_Bi_2_Br_9_ and AgBr as plausible degradation products. Asterisks in the recycled sample denote the peaks ascribable to the AgBr phase in the recovered material.

To assess recyclability, the catalyst was recovered after each run and reused in up to five consecutive cycles of TMB bromination. While the catalytic performance remained unperturbed across all cycles, XRD pattern of the recovered material confirmed the preservation of the structural integrity of the pristine catalyst with a minor impurity phase imputable to AgBr (Figure 5), likely due to surface bromide modification during repeated catalysis cycles.

**FIGURE 6:**
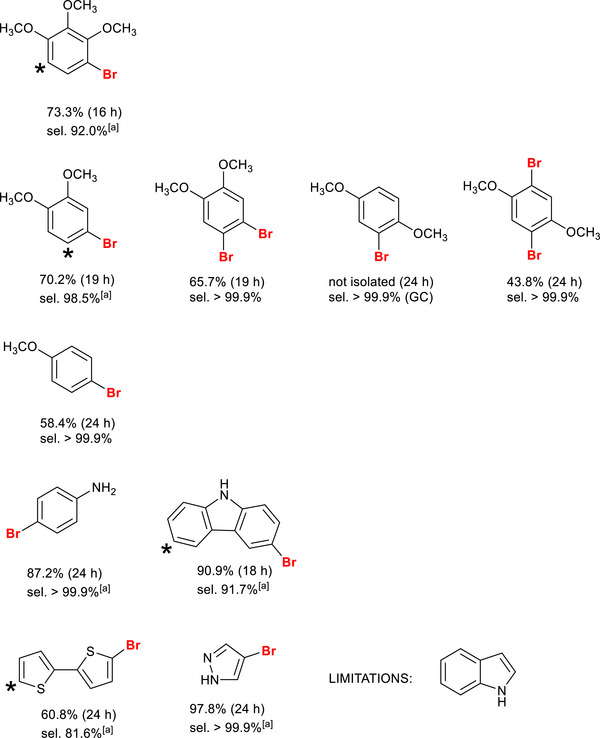
Chemical structure (with the relevant yields and selectivity ascertained by ^1^H NMR) of the substrates utilized with this methodology. For each product is indicated the isolated yield (considering the average molecular weight of the products), reaction time (in the brackets) and selectivity evaluated by ^1^H NMR. ^[a]^Contamination from dibromo‐derivatives (Figures S7–S16). *Indicates the position of the second bromine atom in the formed byproducts.

The methodology proved to be effective across a range of substrates predisposed to bromination. A systematic investigation was conducted to assess the impact of both the position of methoxy substituents on the aryl ring and the number of activating groups (Figure 6). Bromination of 1,2,3‐trimethoxybenzene occurred selectively at the 4‐position, consistent with the electronic orientation imparted by the substituents, while only traces (8.0%) of the 4,6‐dibromo derivative formed under these conditions. The lower chemoselectivity of this substrate can be explained by the lower steric constraints encountered by entering bromine in *ortho* to the methoxy groups. In the case of 1,2‐dimethoxybenzene, the mono‐bromo functionalization at the 4‐position was more selective (98.5%), which is accompanied only by traces of the dibromo product. This substrate undergoes a second, regioselective bromination (> 99.9%) upon treatment with 2 equivalents of HBr, selectively yielding the 4,5‐dibrominated product. In 1,4‐dimethoxybenzene, bromination is directed in *ortho* to the methoxy groups, and dibromination is likewise achievable. However, this organic scaffold was revealed to be unstable with a marked predisposition to oxidation, forming the corresponding quinoidal compounds. An excess HBr selectively led to the isolation of the 2,5‐dibromo derivative, although with relatively low yields for the above‐mentioned side reactions. A similar regioselectivity pattern was observed with anisole with the selective functionalization (> 99.9%) of the *para* position. The methodology was further extended to aromatic substrates bearing different activating groups, such as nitrogen‐containing systems (e.g., aniline and carbazole), as well as five‐membered heterocycles (e.g., 2,2′‐bithiophene, pyrazole, and indole). Regioselective bromination of aniline was observed, with exclusive substitution at the para position. In the case of carbazole, bromination proceeded with slightly lower regioselectivity (91.7%), leading to the formation of a minor amount of the corresponding dibrominated by‐product. For 2,2′‐bithiophene, functionalization of the substrate was confirmed by the detection of the dibrominated species in approximately 20% yield. Pyrazole underwent highly regioselective bromination at the 4‐position, affording the desired product in excellent yield (97.8%). Conversely, the protocol proved ineffective for indole: although the brominated derivative was detected by GC–MS during reaction monitoring, no isolable product was obtained at the end of the reaction, likely due to degradation of the indole‐based scaffold in the presence of the catalyst under the reaction conditions. The variation in isolated yields across different substrates is mainly due to their lower electronic activation compared to TMB, which increases reaction time and promotes side reactions typical of radical processes.[35]

## 3 Conclusions

In this work, we have established a sustainable and atom‐efficient photocatalytic platform for the bromination of electron‐rich arenes, enabled by Cs_2_AgBiBr_6_ microparticles as a class of low‐toxicity double perovskites. This photocatalyst outperforms established perovskite‐like semiconductors such as CsPbBr_3_ and Cs_3_Bi_2_Br_9_, delivering superior yields, regioselectivity, and operational robustness under remarkably mild conditions. Using hydrobromic acid as a benign and atom‐economical bromine source, the system affords mono‐brominated products with selectivity exceeding 99.9%, while maintaining high catalytic productivity. Mechanistic experiments reveal a previously unrecognized activation mode. Bromine radicals central to the catalytic cycle originate from the Cs_2_AgBiBr_6_ surface, which donates lattice bromide upon photoexcitation and is subsequently regenerated by external HBr. This dual bromine‐transfer mechanism (surface donation followed by halide replenishment) provides the first evidence that double perovskites can actively participate in bond activation via sacrificial and restorative surface chemistry. Radical‐trapping studies, degradation patterns observed in bromide‐free conditions, and the absence of peroxide‐based oxidation pathways collectively support this unprecedented mechanism. Furthermore, the catalyst maintains its structural integrity over multiple cycles, with only minor formation of AgBr, highlighting its practical robustness. The methodology exhibits broad functional‐group tolerance and predictable regioselectivity across various aromatic and heteroaromatic substrates, including polymethoxylated arenes, anisole derivatives, bithiophene, and carbazole. Differences in yield and selectivity across the substrate scope mirror their intrinsic electronic properties and the radical nature of the transformation. Overall, this study demonstrates that halide double perovskites can function not merely as passive light absorbers but as dynamic photocatalytic platforms in which lattice halides serve as active participants in redox processes. These findings open new perspectives for exploiting perovskite surface reactivity in synthetic photocatalysis and suggest promising opportunities for the development of greener halogenation methodologies and beyond.

## 4 Experimental Section

### 4.1 Materials and Methods

Cesium bromide (CsBr, 99.999% trace metals basis, Sigma‐Aldrich), bismuth(III) bromide (BiBr_3_, ≥ 98%, Sigma‐Aldrich), lead(II) bromide (PbBr_2_, 99.999% trace metals basis, Sigma‐Aldrich), silver(I) bromide (AgBr, ≥ 99.0%, Sigma‐Aldrich), biphenyl (BP, ≥ 99.0%, Sigma‐Aldrich), dimethyl sulfoxide (DMSO, anhydrous, ≥ 99.9%, Sigma‐Aldrich), 2‐propanol (IPA, ≥ 99.8%, Honeywell Riedel‐de Haën), acetonitrile (ACN, ≥ 99.9%, GC grade, Sigma‐Aldrich) and hydrobromic acid (HBr, 47 wt% in water, Sigma‐Aldrich) were used as received. Titanium(IV) oxysulfate solution (TiOSO_4_, technical grade, ≥ 29% TiO_2_ basis, Sigma‐Aldrich), hydrogen peroxide solution (H_2_O_2_, ACS reagent, ≥ 30%, Sigma‐Aldrich), and sulfuric acid (H_2_SO_4_, 96%, ultrapure, Sigma‐Aldrich) were employed for photocatalytic and colorimetric detection of H_2_O_2_ via TiOSO_4_ complexation in acidic medium. Distilled water (Grade II) was used throughout for aqueous preparations and titanyl‐based colorimetric detection of H_2_O_2_. 1,3,5‐trimethoxybenzene, 1,2,3‐trimethoxybenzene, 1,2‐dimethoxybenzene, 1,4‐dimethoxybenzene, anisole, 2,2′‐bithiophene, carbazole—all ≥ 98%, purchased from Sigma‐Aldrich and used as received. 2,2,6,6‐tetramethylpiperidin‐1‐oxyl (TEMPO, ≥ 98%, Sigma‐Aldrich) was used as received. The morphology of the powders was acquired by SEM coupled with energy dispersive X‐ray spectroscopy using a Zeiss Σigma 300VP electron microscope equipped with an Oxford C‐MaxN SDD detector with an active area of 20 mm^2^. Perovskite samples were deposited on aluminum stubs, and the images were recorded at working distance of 7.5 mm, an acceleration voltage of 15 kV and a magnification of 1000×. The analysis accuracy was checked using the MAC (Micro‐Analysis Consultants Ltd) reference materials. XPS analysis was conducted using an ultrahigh vacuum (UHV) system. Prior to measurement, Cs_2_AgBiBr_6_ particles were deposited onto doubles sided Cu tape and allowed to degas overnight in a vacuum desiccator. The XPS spectra were collected using a twin anode X‐ray source (8025 Twin Anode, V.G. Microtech) operating with nonmonochromatized Al Kα_1,2_ radiation (photon energy = 1486.6 eV), coupled with a hemispherical electron energy analyzer (CLAM4 MCD LNo5, V.G. Microtech). Data acquisition and analysis were performed using CasaXPS software (version 2.3.25 PR1.0). Prior to peak fitting, a Shirley background subtraction was applied. The spectral features were fitted using a Gaussian–Lorentzian function to model the line shapes of the components. All spectra were calibrated by referencing the C 1s (C–C) peak to 284.8 eV. UV–Vis diffuse reflectance spectroscopy was conducted using a Jasco V670 spectrophotometer equipped with an integrating sphere. Electrochemical measurements were performed using a Metrohm Autolab PGSTAT 302‐N potentiostat in acetonitrile (0.10 M n‐Bu_4_PF_6_) at a scan rate of 100 mV/s in a three‐electrode configuration. Diffractograms were recorded using a Malvern Panalytical Empyrean α 1 in a powder diffraction mode using Cu Kα radiation (*λ* = 1.5406 Å) with a cathode voltage and current of 45 kV and 40 mA, respectively (range: 10−60°, step size: 0.0262°, time per step: 17 s). The diffraction pattern was collected immediately in ambient conditions (T = 22°C, relative humidity = 30%). The substrate conversion and the product evolution were determined by GC–MS (EI, 70 eV) performed on an HP 6890 instrument equipped with an HP‐5MS 5% phenyl methyl siloxane (30.0 m × 250 µm × 0.25 µm) coupled with an HP 5973 mass spectrometer passing from 50°C to 280°C with a ramp of 15°C/min. ^1^H‐NMR spectra were recorded at 300 MHz on a Bruker Avance DPX 300 MHz spectrometer using standard pulse sequences. Samples were dissolved in acetone‐d_6_, chemical shifts (δ) are reported in ppm and referenced to the residual solvent peaks (δ = 2.05 ppm for acetone‐d_6_).

### 4.2 Synthesis

The double perovskite Cs_2_AgBiBr_6_ was synthesized following an analogous protocol. CsBr (0.45 mmol, 96.0 mg), BiBr_3_ (0.225 mmol, 100.96 mg), and AgBr (0.225 mmol, 42.25 mg) were dissolved in anhydrous DMSO (10 mL) under stirring at room temperature. The homogeneous solution was promptly injected into isopropanol (200 mL) under vigorous agitation, yielding a pale‐yellow solid. The precipitate was collected by centrifugation (4000 rpm, 10 min), thoroughly washed with ethanol (3 × 20 mL), and dried under vacuum at 50°C for 24 h. The final product was obtained in 187 mg yield. CsPbBr_3_ was prepared via a tailored synthetic route developed in our laboratory based on previously reported methods with minor modification, by dissolving CsBr (0.45 mmol, 96.0 mg) and PbBr_2_ (0.45 mmol, 165.15 mg) in anhydrous DMSO (10 mL) under magnetic stirring at room temperature. The clear solution was rapidly injected into isopropanol (200 mL) under vigorous stirring. A bright yellow solid formed immediately, which was collected by centrifugation (4000 rpm, 10 min), washed with ethanol (3 × 20 mL), and vacuum‐dried at 50°C for 24 h. The yield was 190 mg. The bismuth‐based perovskite Cs_3_Bi_2_Br_9_ was prepared via an antisolvent reprecipitation approach. CsBr (0.45 mmol, 96.0 mg) and BiBr_3_ (0.30 mmol, 134.5 mg) were dissolved in anhydrous dimethyl sulfoxide (DMSO, 10 mL) under constant stirring at room temperature. The resulting clear solution was rapidly injected into vigorously stirred isopropanol (200 mL), resulting in the immediate formation of a yellow precipitate. The solid was collected by centrifugation (4000 rpm, 10 min), washed with ethanol (3 × 20 mL) to remove residual reagents and solvent, and dried under vacuum at 50 °C for 24 h. The final yield was 183 mg.

### 4.3 Electrochemical Characterization

CV measurements were performed in dry acetonitrile containing 0.1 M n‐Bu_4_NPF_6_ as the supporting electrolyte, using a conventional three‐electrode configuration. A glassy carbon disk (3 mm diameter) served as the working electrode, a platinum wire as the counter electrode, and a silver wire was used as a pseudo‐reference electrode. All measurements were carried out under a nitrogen atmosphere. Ferrocene (Fc, 1.0 mM) was added to each solution as an internal standard for potential calibration. The oxidation onset of 1,3,5‐trimethoxybenzene (TMB, 1.0 mM) was recorded at +0.90 V versus the Fc/Fc^+^ couple. Ferrocene displayed an onset at +0.45 V under identical conditions. Using the accepted value of +0.63 V for the Fc/Fc^+^ couple versus NHE in acetonitrile, the onset potentials were converted to the NHE scale. On this basis, the oxidation onset potentials versus NHE were calculated to be +1.53 V for TMB, +1.15 V for HBr, and +1.00 V for Cs_2_AgBiBr_6_. The HBr measurement was conducted by adding a 47% aqueous solution to acetonitrile under the same electrolyte conditions, ensuring homogeneous mixing. The Cs_2_AgBiBr_6_ film was prepared by drop‐casting a suspension of the powdered material in ethanol onto the glassy carbon electrode, followed by drying in air. To evaluate the feasibility of oxygen activation under the applied photocatalytic conditions, the reduction of molecular oxygen was also investigated electrochemically under identical conditions. CV was recorded in air‐saturated acetonitrile containing 0.1 M *n*‐Bu_4_NPF_6_ at the glassy carbon electrode. A clear reduction peak was observed at −0.64 V versus Ag/Ag^+^, which corresponds to −1.10 V versus Fc/Fc^+^ (see Figure S3), and thus to −0.46 V versus NHE. This value is consistent with the literature range for the $O_{2}/O_{2}^{-\cdot }$ redox couple in aprotic media and indicates that the photogenerated electrons from the conduction band of Cs_2_AgBiBr_6_ (estimated at ca. −1.26 V vs. NHE) possess sufficient reducing power to activate molecular oxygen via single‐electron transfer.

### 4.4 Photocatalysis

Photocatalytic bromination was carried out in 5 mL borosilicate glass tubes (12 mm diameter, 75 mm length) containing TMB (0.04 mmol, 6.71 mg), HBr (0.05 mmol, 5.6 µl), acetonitrile (3.0 mL), and Cs_2_AgBiBr_6_ (3.00 mg, 6% mol/mol) and biphenyl as internal standard (0.10 mmol, 15.40 mg). The reaction mixture was stirred and irradiated using a 3 W blue LED placed at a fixed distance of 10 cm from the sample. Product yields and conversions were determined by GC‐MS analyses using biphenyl as an internal standard. In the case of isolated yields, after complete conversion determined by GC analyses, the catalyst was removed by centrifugation. The supernatant was concentrated under reduced pressure, and the crude mixture was purified by flash column chromatography using *n*‐hexane/ethyl acetate (9:1 vol:vol) as an eluent. In the case of stability study, after the standard reaction (entry 1 of Table 1) to full substrate conversion, the catalyst was recovered by centrifugation, washed (3× with acetonitrile, then with ethanol), dried under vacuum at 60°C for 24 h, and reused for a total of five cycles.

### 4.5 Radical Scavengers

To ascertain the radical nature of the reaction pathway, selected experiments of Table 1 of the manuscript were conducted using TEMPO as a radical scavenger. Following the conditions of entry 4 (Table 1), the reaction of TMB (0.04 mmol) with Cs_2_AgBiBr_6_ (3 mg) in acetonitrile (3.0 mL) was carried out in the presence of TEMPO (0.40 mmol, 62.5 mg). After 24 h of irradiation, the reaction mixture was centrifuged, filtered, and the supernatant analyzed by GC–MS. The recovered photocatalyst appeared with the same consistency of the material recovered after the reaction of entry 4 of Table 1, indicating the evolution of bromine radicals from the catalyst, trapped by the scavenger. In a parallel experiment, the standard reaction (entry 1 of Table 1) was carried out in the presence of TEMPO (0.40 mmol, 62.5 mg).

### 4.6 Qualitative Detection of Hydrogen Peroxide

A 0.078 M solution of titanium(IV) oxysulfate in 0.2 M sulfuric acid was prepared by dissolving titanium(IV) oxysulfate in 0.2 M aqueous sulfuric acid under gentle heating. The resulting solution was clear and colorless and stored in an amber bottle protected from light at 4°C. A 1.0 mM hydrogen peroxide standard solution was freshly prepared by dilution of commercial 30% H_2_O_2_ with ultrapure water immediately prior to use. For qualitative analysis, 2.0 mL of the titanium oxysulfate solution was mixed with 1.0 mL of the sample solution in a 3.0 mL quartz cuvette. The appearance of a yellow coloration and the formation of a characteristic absorption band centered around 405 nm in the UV‐vis spectrum indicate the presence of hydrogen peroxide via the formation of the peroxotitanium complex.

## Conflicts of Interest

The authors declare no conflicts of interest.

## Supporting information




**Supporting File 1**: chem70837‐sup‐0001‐SuppMat.docx.

## Data Availability

The data that support the findings of this study are available in the supplementary material of this article.
