# Supplementary material for: Insights Into the Photocatalytic Arene Bromination Enabled by Cs2AgBiBr6 Microparticles
Source: Chemistry. 2026 Feb 28;32(18):e70837. doi: 10.1002/chem.70837 (PMC13174901; doi:10.1002/chem.70837)
Supplement: Supplementary file 1 — Supporting File 1: chem70837‐sup‐0001‐SuppMat.docx. [file CHEM-32-e70837-s001.docx]

**Supporting Information**

Insights into the photocatalytic arene bromination enabled by Cs_2_AgBiBr_6_ microparticles

Daniele Conelli,^[a]^ Chiara Lo Porto, ^[b]^ Mokurala Krishnaiah,^[c]^ Kimmo Lahtonen,^[c]^ G. Krishnamurthy Grandhi,^[c]^ Nicola Margiotta,^[d]^ Paola Vivo,^[c]^ Gian Paolo Suranna^[a,e]^ and Roberto Grisorio*^[a]^

^[a]^Dipartimento di Ingegneria Civile, Ambientale, del Territorio, Edile e di Chimica (DICATECh), Politecnico di Bari, Via Orabona 4, 70125 Bari, Italy. E-mail: roberto.grisorio@poliba.it

^[b]^Department of Life Science, Health, and Health Professions, Università degli Studi Link, via del Casale di San Pio V, 44, 00165 Roma, Italy.

^[c]^Hybrid Solar Cells, Faculty of Engineering and Natural Sciences, P.O. Box 541, FI-33014 Tampere University, Finland.

^[d]^Dipartimento di Chimica, Università degli Studi di Bari “Aldo Moro”, via Orabona 4, 70125 Bari, Italy.

^[e]^CNR-NANOTEC – Institute of Nanotechnology, c/o Campus Ecoteckne, Via Monteroni, 73100 Lecce, Italy.

***Figure S1.*** *(a) Survey XPS spectrum of the Cs_2_AgBiBr_6_ samples, and (b) high-resolution XPS spectrum of C 1s.*

***Figure S2.*** *Kubelka–Munk plot used for the direct bandgap estimation. The larger value indicates the indirect nature of the electronic transitions in the Cs_2_AgBiBr_6_ microparticles of this study.*

**

***Figure S3.*** *Cyclic voltammograms recorded in acetonitrile containing 0.1 M n-Bu_4_NPF_6_ under O_2_ (blue trace, O_2_/O_2_⁻ redox couple) and N_2_ atmospheres (black trace, reference scan). The N_2_ scan serves to highlight background currents in the absence of oxygen.*


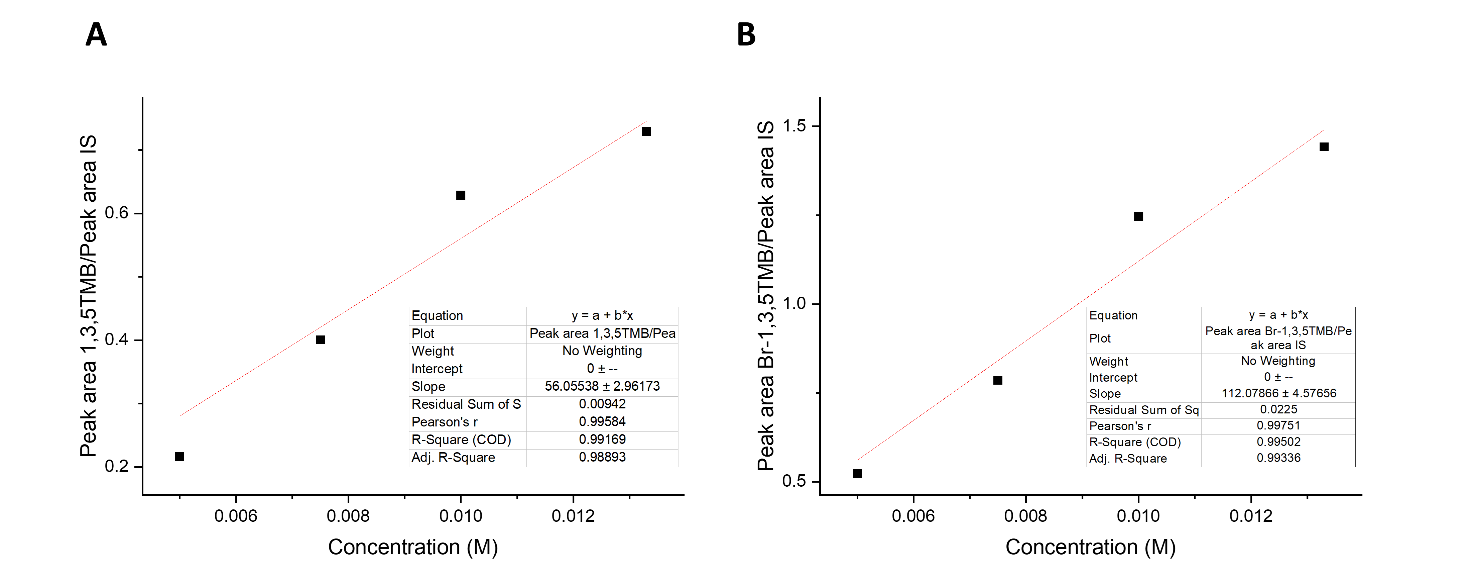


***Figure S4.*** *Calibration curves used for GC-based quantification. (A) Plot of peak area ratio (1,3,5-trimethoxybenzene/biphenyl) vs. molar concentration. (B) Plot of peak area ratio (2-bromo-1,3,5-trimethoxybenzene/biphenyl) vs. molar concentration. Calibration was performed using biphenyl as internal standard; linear regression details are shown in the insets.*


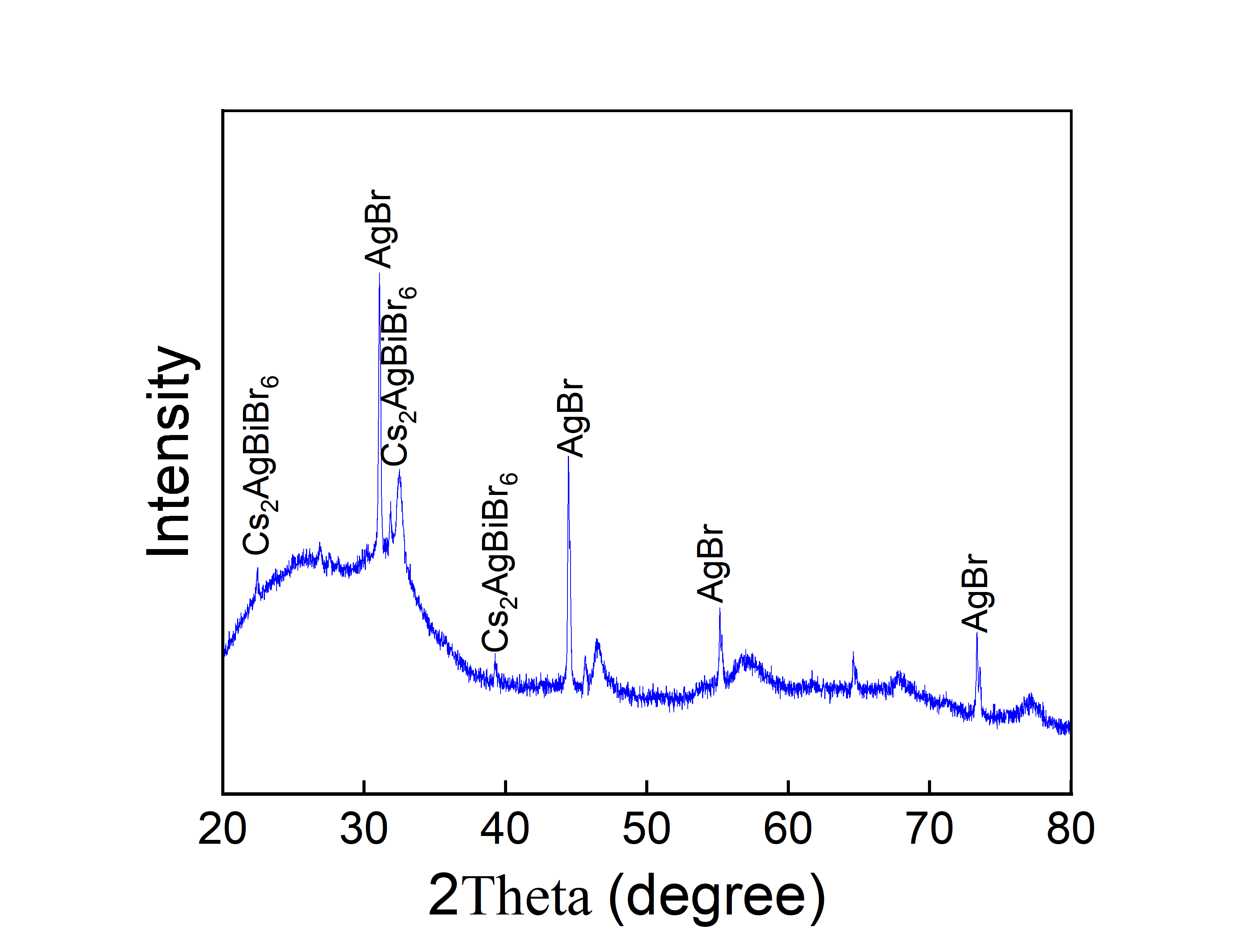


***Figure S5.*** *XRD analysis of the Cs_2_AgBiBr_6_ material recovered after the reaction performed without HBr (entry 4 of Table 1 of the manuscript).*

***
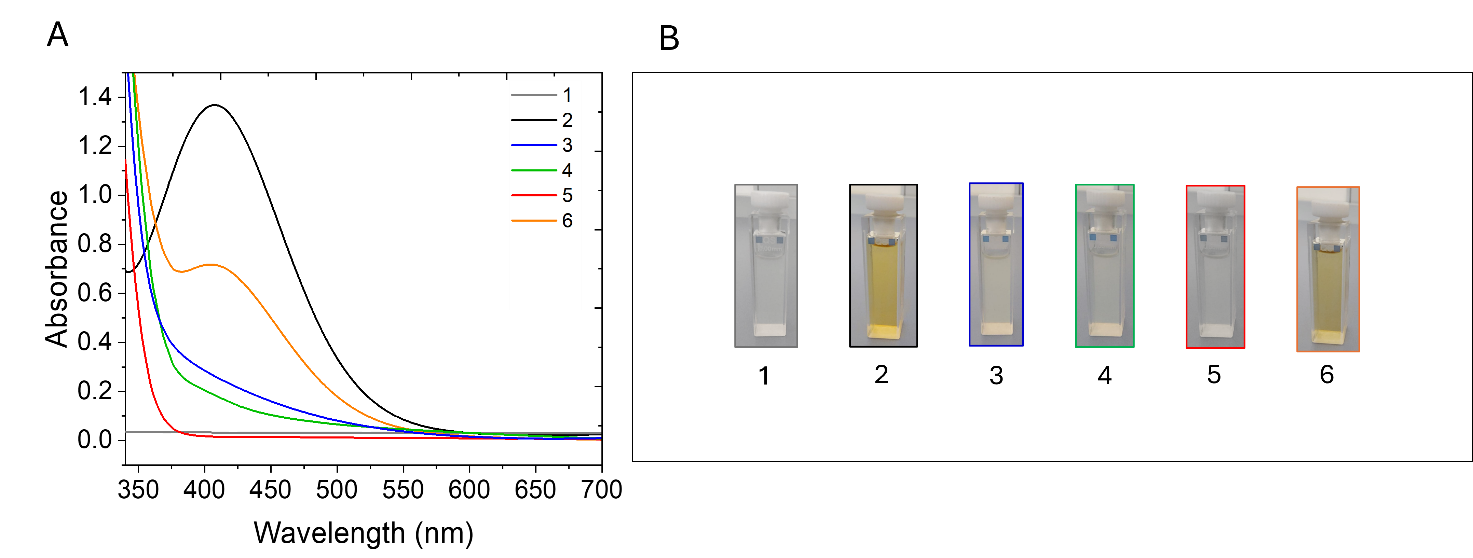
***

***Figure S6.*** *(A) UV-vis spectra showing qualitative detection of hydrogen peroxide via formation of a yellow peroxotitanium complex with acidic TiOSO_4_ (λ_max_ ≈ 405 nm). (B) Corresponding cuvettes photographed under ambient light. From left to right: (1) blank (TiOSO_4_ only), (2) TiOSO_4_ + H_2_O_2_ (0.1 mM), (3) TiOSO_4_ + reaction mixture (TMB + HBr + photocatalyst), (4) TiOSO_4_ + reaction mixture without HBr (TMB + photocatalyst), (5) TiOSO_4_ + reaction mixture without photocatalyst (TMB + HBr), (6) TiOSO_4_ + reaction mixture without photocatalyst (TMB + HBr) spiked with H_2_O_2_.*


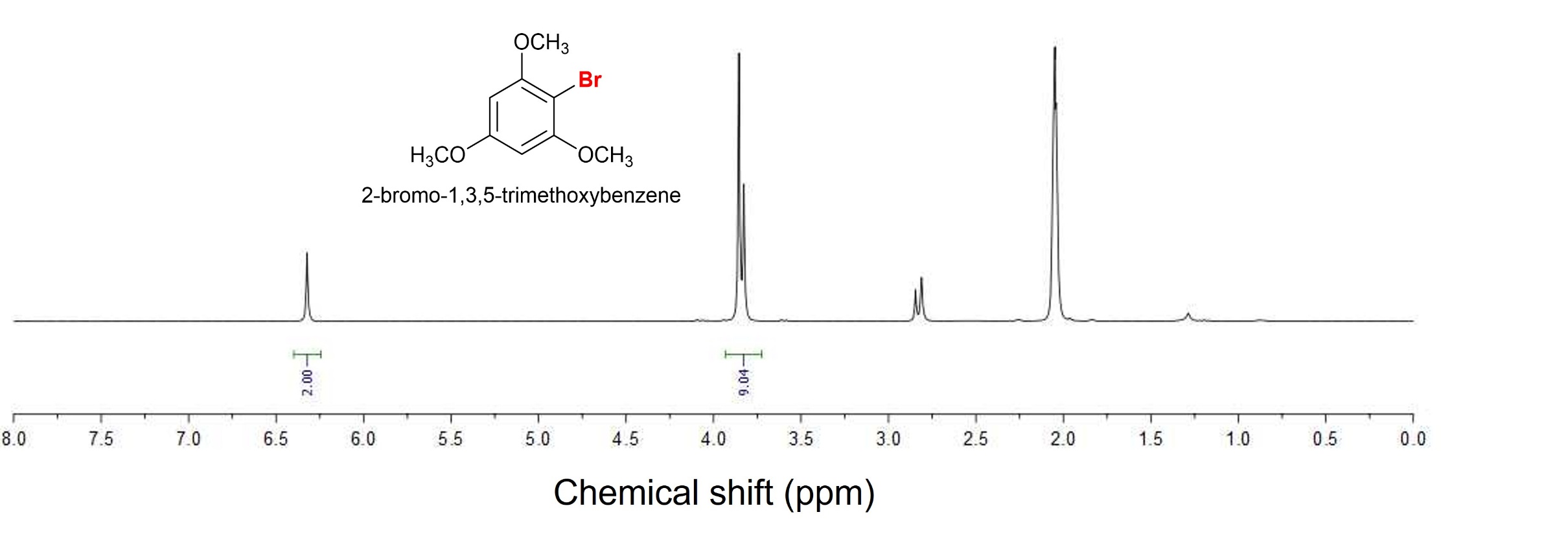


***Figure S7.*** *^1^H NMR spectrum of 2-bromo-1,3,5-trimethoxybenzene recorded in acetone-d_6_ (300 MHz, 298 K).*


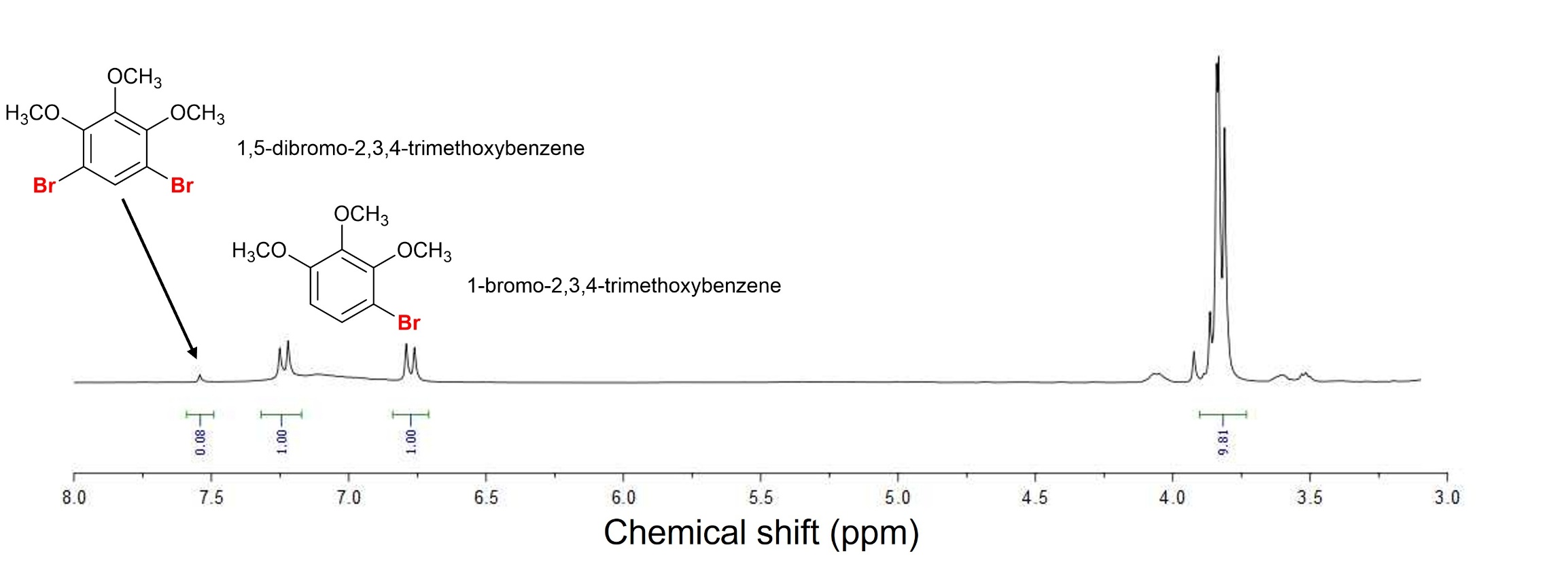


***Figure S8.*** *^1^H NMR spectrum of 1-bromo-2,3,4-trimethoxybenzene recorded in acetone-d_6_ (300 MHz, 298 K).*


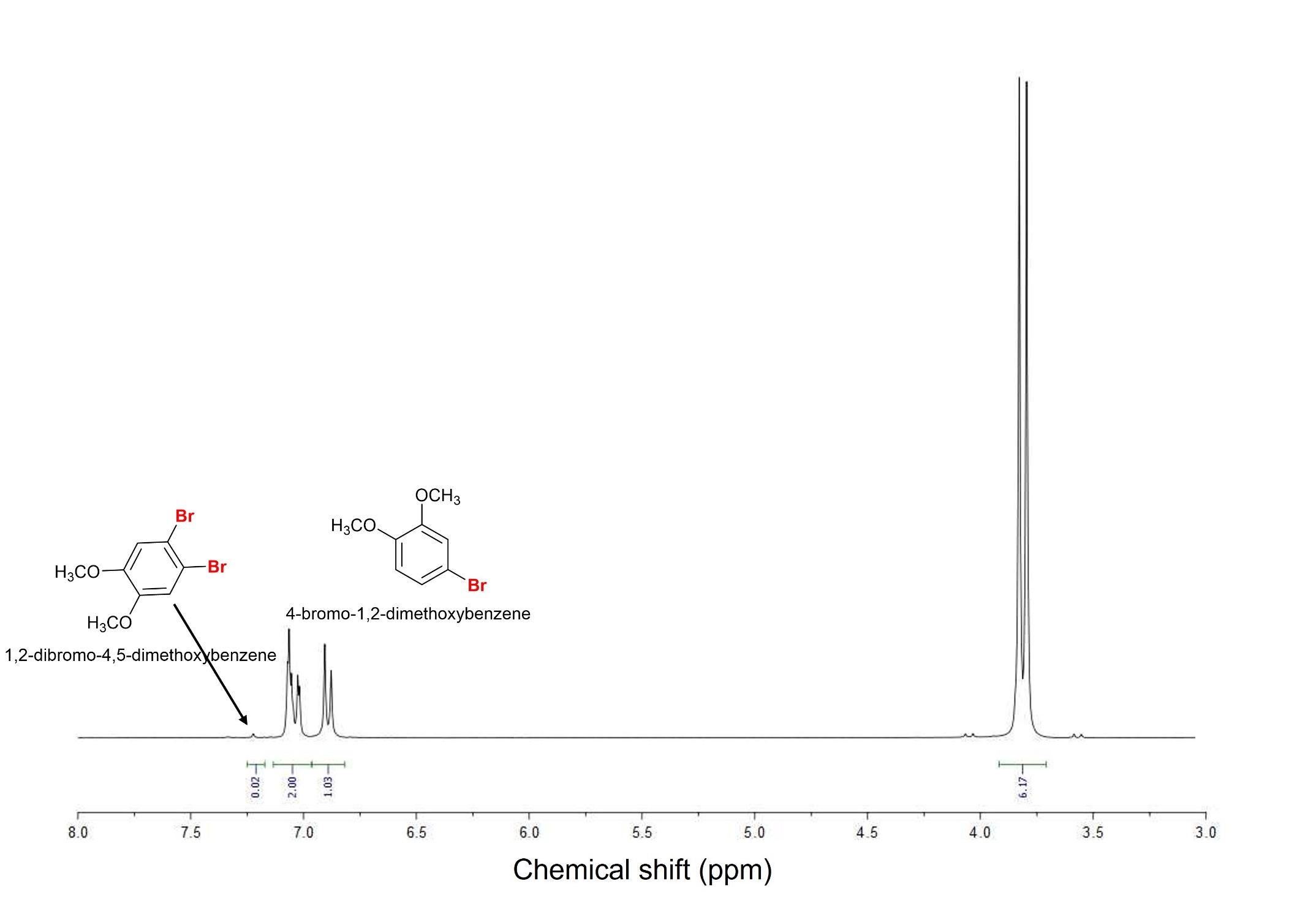


***Figure S9.*** *^1^H NMR spectrum of 4-bromo-1,2-dimethoxybenzene recorded in acetone-d_6_ (300 MHz, 298 K).*


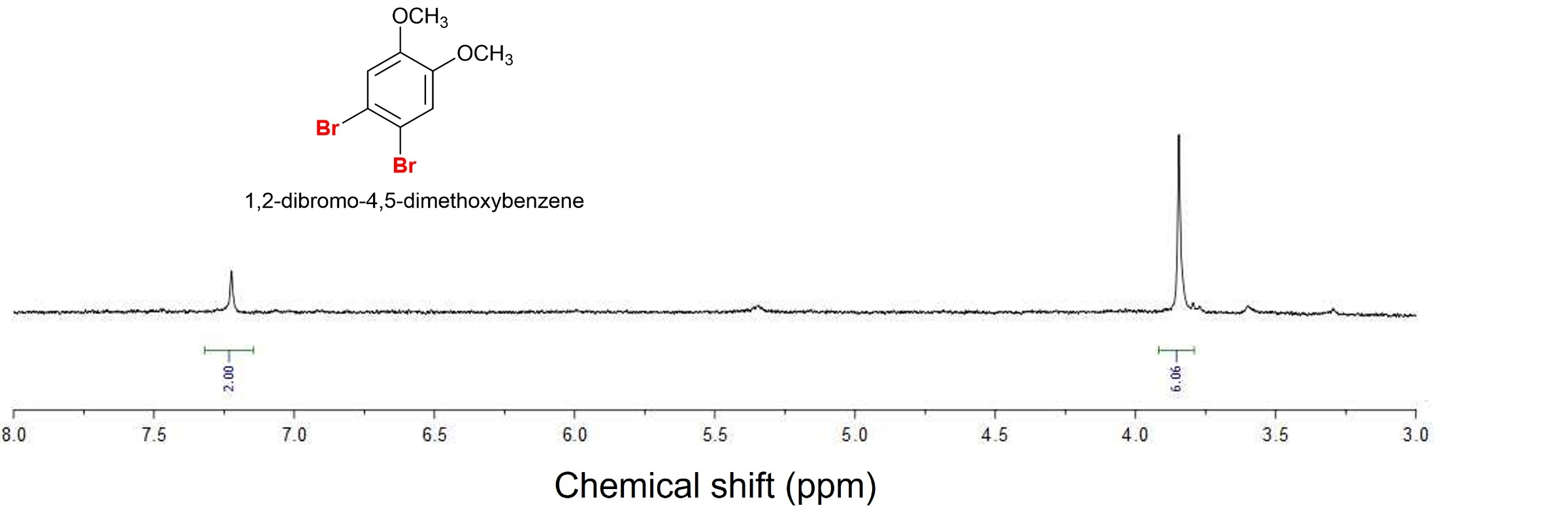


***Figure S10.*** *^1^H NMR spectrum of 1,2-dibromo-4,5-dimethoxybenzene recorded in acetone-d_6_ (300 MHz, 298 K).*


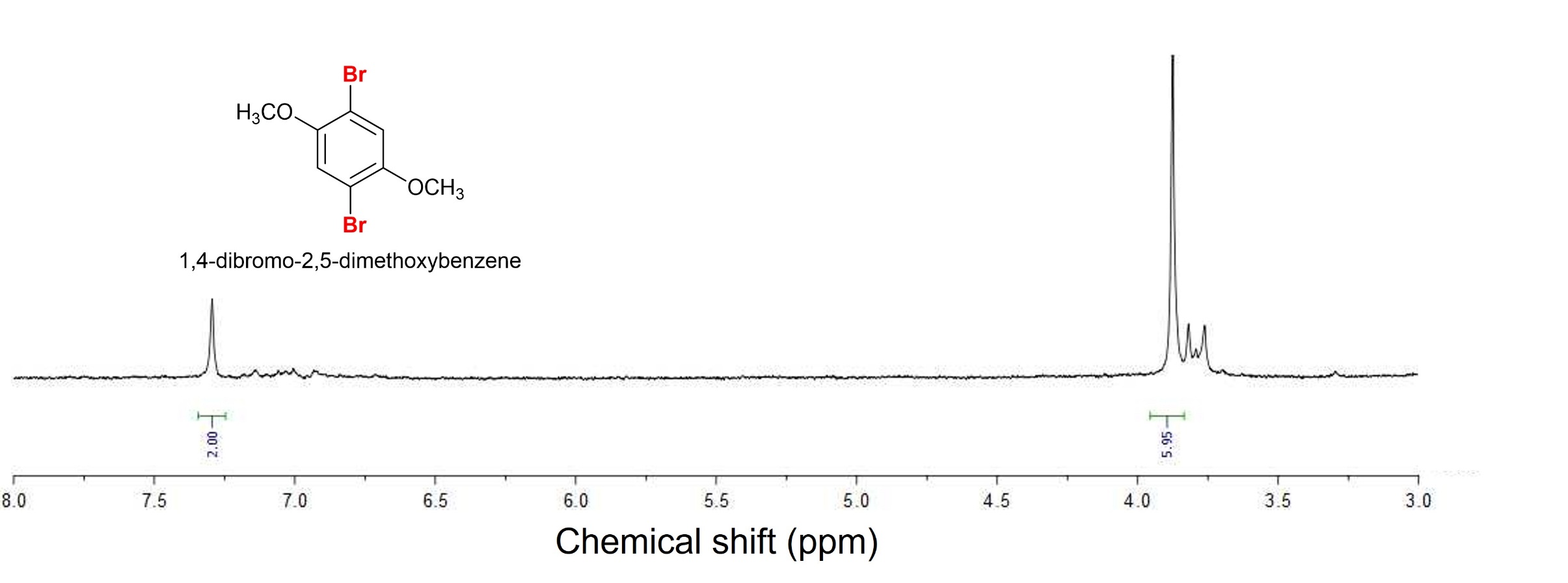


***Figure S11.*** *^1^H NMR spectrum of 1,4-dibromo-2,5-dimethoxybenzene recorded in acetone-d_6_ (300 MHz, 298 K).*


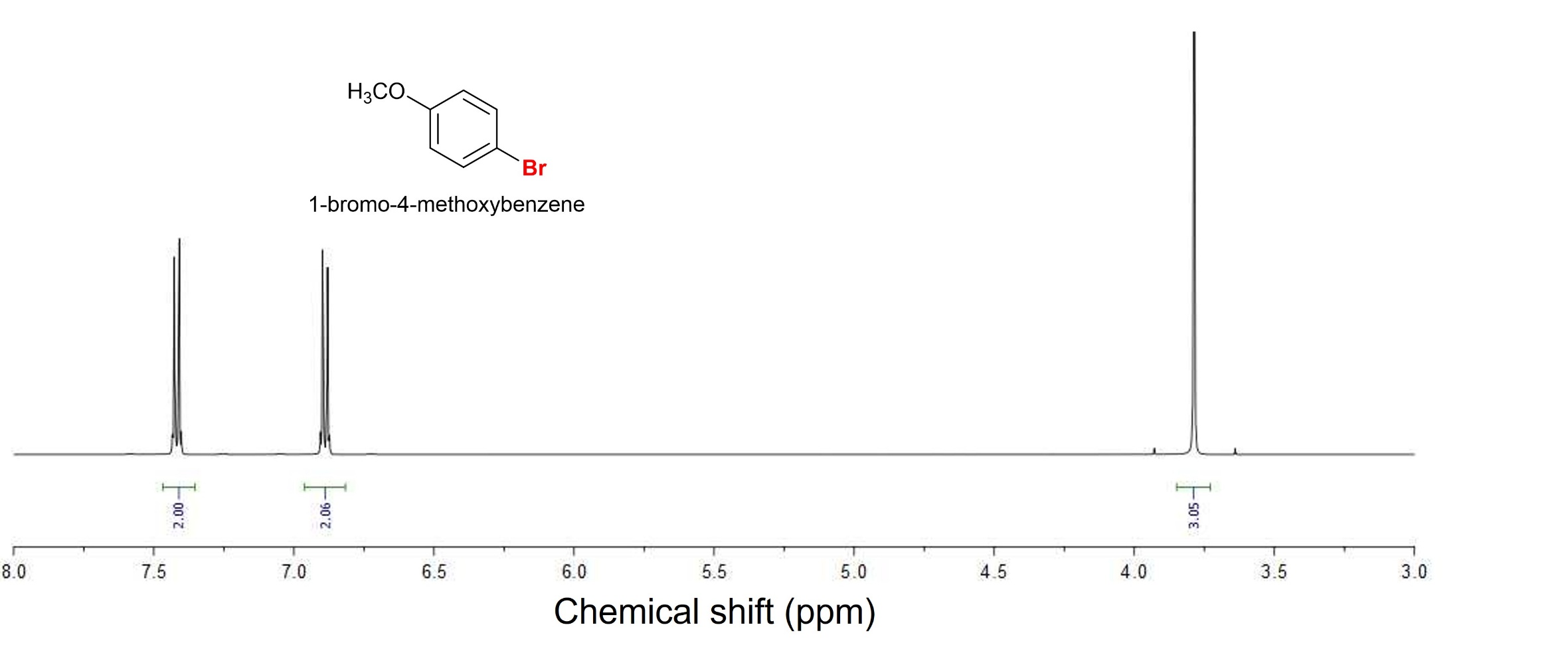


***Figure S12.*** *^1^H NMR spectrum of 1-bromo-4-methoxybenzene recorded in acetone-d_6_ (300 MHz, 298 K).*


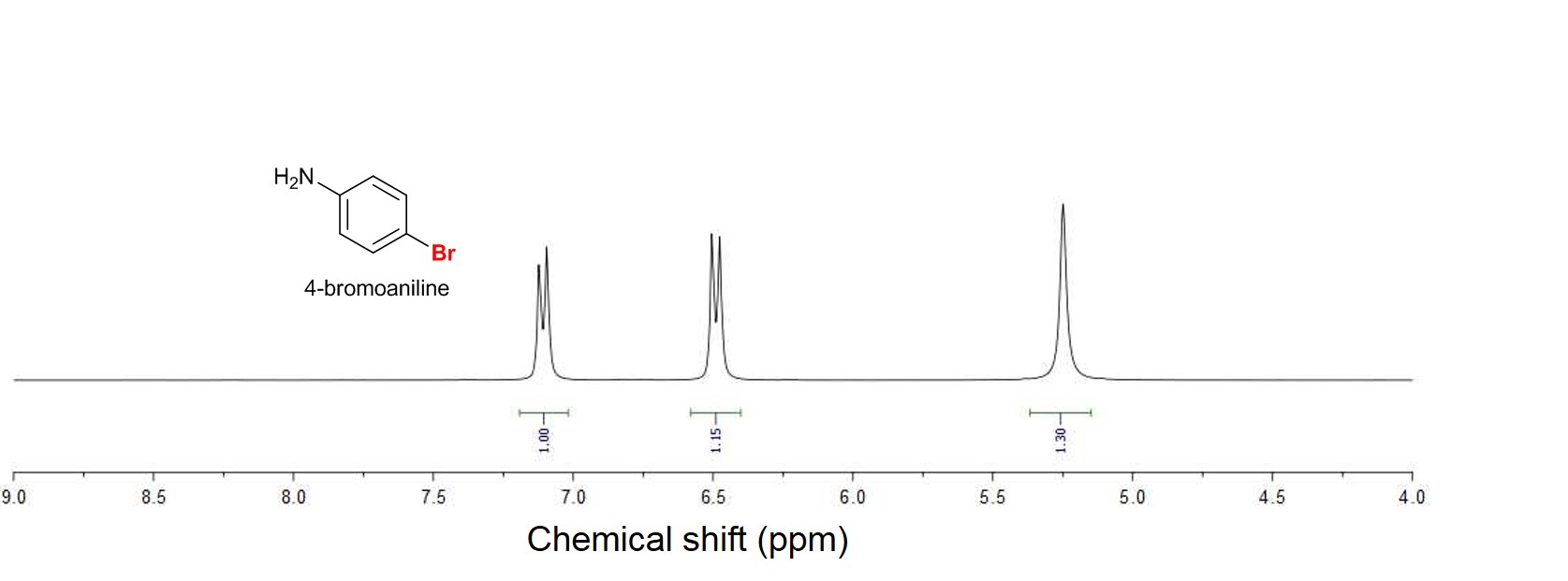


***Figure S13.*** *^1^H NMR spectrum of 4-bromo-aniline recorded in DMSO-d_6_ (300 MHz, 298 K).*

*
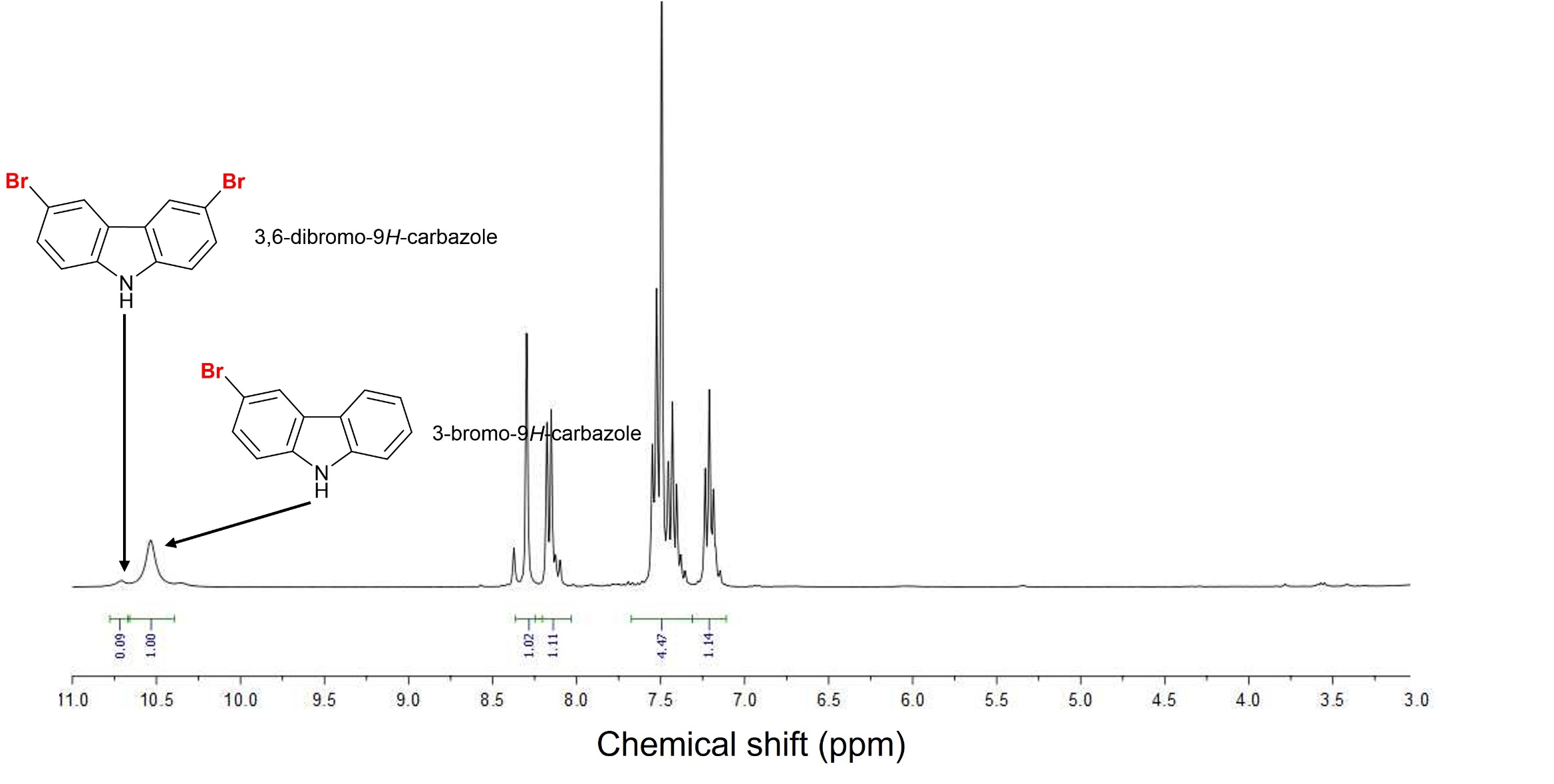
*

***Figure S14.*** *^1^H NMR spectrum of 3-bromo-9H-carbazole recorded in acetone-d_6_ (300 MHz, 298 K).*

*
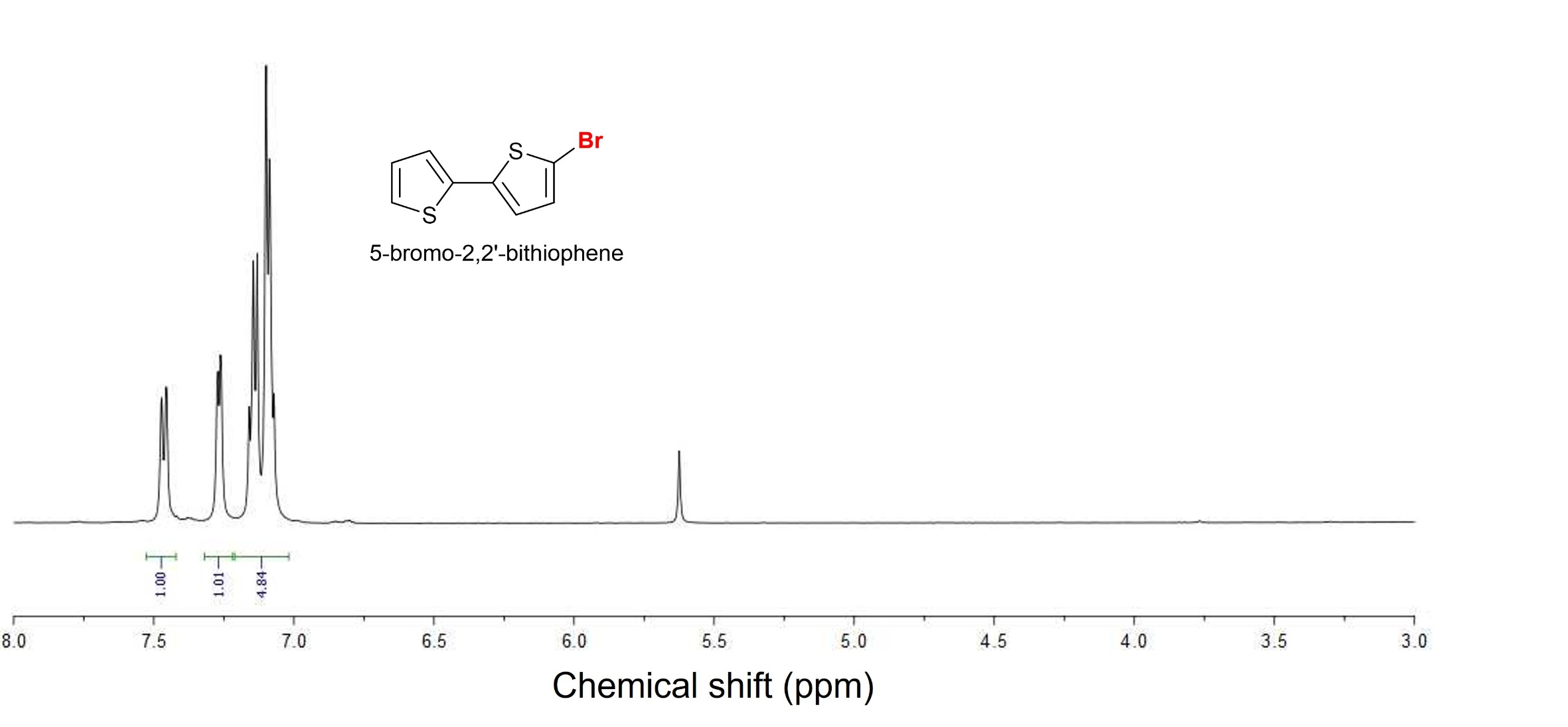
*

***Figure S15.*** *^1^H NMR spectrum of 5-bromo-2,2'-bithiophene recorded in acetone-d_6_ (300 MHz, 298 K).*

*
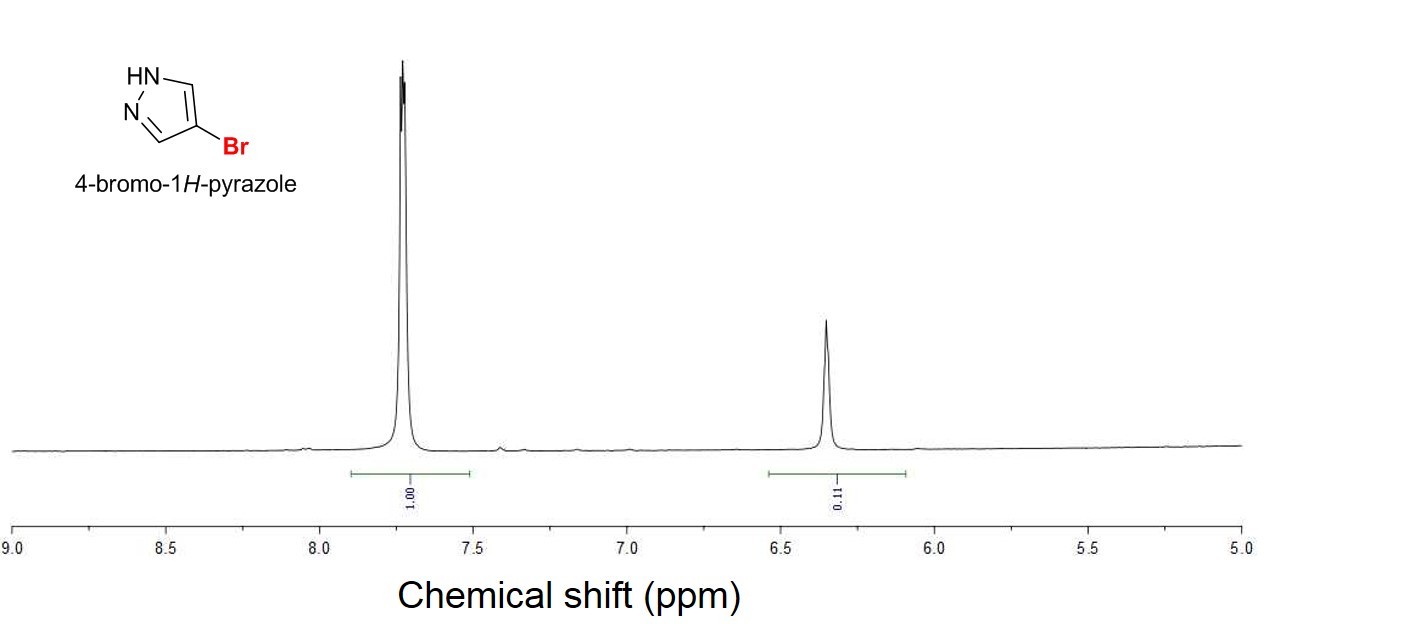
*

***Figure S16.*** *^1^H NMR spectrum of 4-bromo-1H-pyrazole recorded in DMSO-d_6_ (300 MHz, 298 K).*
